# Synaptic Failure Differentially Affects Pattern Formation in Heterogenous Networks

**DOI:** 10.3389/fncir.2019.00031

**Published:** 2019-05-08

**Authors:** Maral Budak, Michal Zochowski

**Affiliations:** ^1^Biophysics Program, University of Michigan, Ann Arbor, MI, United States; ^2^Department of Physics, University of Michigan, Ann Arbor, MI, United States

**Keywords:** synaptic transmission failure, network dynamics, network synchrony, spatio-temporal pattern formation, scale-free networks

## Abstract

The communication of neurons is primarily maintained by synapses, which play a crucial role in the functioning of the nervous system. Therefore, synaptic failure may critically impair information processing in the brain and may underlie many neurodegenerative diseases. A number of studies have suggested that synaptic failure may preferentially target neurons with high connectivity (i.e., network hubs). As a result, the activity of these highly connected neurons can be significantly affected. It has been speculated that anesthetics regulate conscious state by affecting synaptic transmission at these network hubs and subsequently reducing overall coherence in the network activity. In addition, hubs in cortical networks are shown to be more vulnerable to amyloid deposition because of their higher activity within the network, causing decrease in coherence patterns and eventually Alzheimer’s disease (AD). Here, we investigate how synaptic failure can affect spatio-temporal dynamics of scale free networks, having a power law scaling of number of connections per neuron – a relatively few neurons (hubs) with a lot of emanating or incoming connections and many cells with low connectivity. We studied two types of synaptic failure: activity-independent and targeted, activity-dependent synaptic failure. We defined scale-free network structures based on the dominating direction of the connections at the hub neurons: incoming and outgoing. We found that the two structures have significantly different dynamical properties. We show that synaptic failure may not only lead to the loss of coherence but unintuitively also can facilitate its emergence. We show that this is because activity-dependent synaptic failure homogenizes the activity levels in the network creating a dynamical substrate for the observed coherence increase. Obtained results may lead to better understanding of changes in large-scale pattern formation during progression of neuro-degenerative diseases targeting synaptic transmission.

## Introduction

Neurons transmit signals to communicate predominantly via synapses. The synapses may fail to transmit signals due to the depletion of neurotransmitters or external changes in membrane/ion channel activity. The examples of the latter, include interaction of oligomeric Aß or misfolded tau with cell surface receptors, intracellular signaling molecules or scaffold proteins, which leads to the deterioration of synaptic structure and function causing Alzheimer’s disease (AD) (Chen et al., 2019). Another example is an interaction of anesthetics with GABA_A_ or NMDA receptors, or K^+^ channels, causing hyperpolarization, glutamate desensitization or increase in K^+^ conductance at the postsynaptic neuron, respectively (Franks, 2008).

Subsequently it is no surprise that synaptic failure can change functional network connectivity and consequently information processing leading to devastating outcomes. For instance, synaptic failure is part of the cause of most neurodegenerative diseases including AD, Huntington, ALS, and ischemic cerebral damage. In fact, it is the first pathologic event to occur in these diseases, even before the loss of neurons (Wishart et al., 2006). However, synaptic transmission failure may target different components of the network and lead to different consequences in terms of changes of spatio-temporal patterning in the network. Buckner et al. (2009) provided evidence that cortical hubs (i.e., regions that integrate and transmit information from/to many other parts of the brain) in humans are the most vulnerable areas to amyloid deposition, which results in atrophy and eventually AD. Moreover, another study on mice showed that amyloid deposition is caused by excessive neuronal and synaptic activity *in vivo* (Bero et al., 2011). de Haan et al. (2012) hypothesized that hubs are the most active regions in the brain, resulting in “activity dependent degeneration”. Consequently, they showed that the hubs are the most active regions in the brain and activity dependent degeneration results in hub vulnerability as well as macro-scale disruption of brain connectivity, as observed in AD.

Another hypothetical consequence of synaptic failure is the loss of consciousness via application of anesthetics. Anesthetics are thought to act through ion channel blockage and/or changes in cellular membrane dynamics which lead to synaptic failure (Diao et al., 2014). One of the observed outcomes of anesthetics on a macro-network scale is a decrease in the large-scale functional connectivity between different parts of the brain. In particular, it was postulated that the hub regions of the brain are primarily affected by anesthetics and lead to the loss of the global functional connectivity which is followed by the loss of consciousness (Lee et al., 2013). In a similar spirit, another study investigated directionality of information flow in the network by simulating simple oscillatory models in a human anatomical network. They found the directionality of a network is determined by its topology (Moon et al., 2015). Since the hub nodes phase lag and peripheral nodes phase lead, they concluded that connections are from less to more degree nodes. Further, they perturbed the hub structure to simulate unconscious state, leading to the elimination of the directionality in the neuroanatomical network, which is consistent with anesthetic administered human data, where anterior (less hubs)-to-posterior (more hubs) directionality was lost.

Previously, a theoretical study was done to investigate how the topology of neuronal networks influences their dynamics when they suffer from synaptic loss. In this study, synapses were removed with a given probability, and they observed that bimodal networks are more robust than random ones (Mirzakhalili et al., 2017).

Here, we systematically studied the effects of gradual, stochastic synaptic failure on a functional network connectivity in scale-free networks. The aim of this study is to investigate the universal patterns of changes in functional connectivity based on the pattern and degree of synaptic failure. We specifically, wanted to know how the network responds when neurons having different number of connections (i.e., playing different roles in the network) are targeted. We investigate two modes of synaptic transmission loss: (1) in activity-independent case, transmission probability remains constant for all synapses in the network throughout the simulation, and (2) in activity-dependent case, synapses are more likely to fail, if the postsynaptic neuron has fired more recently. Further, we used both incoming (i.e., hubs predominantly receive the signals) and outgoing (i.e., hubs send signals) networks, since a recent study showed that direction of information flow is not always into the hubs, but can be bi-directional depending on the frequency of the signal (Hillebrand et al., 2016). We assessed the network-wide activity patterns through the degree of synchrony or coherence among the networks. We show that the two studied modes of synaptic failure can lead to non-trivial behavior of the network, which in turn can affect information processing.

## Materials and Methods

### Network Structure and Connectivity

**FIGURE 1:**
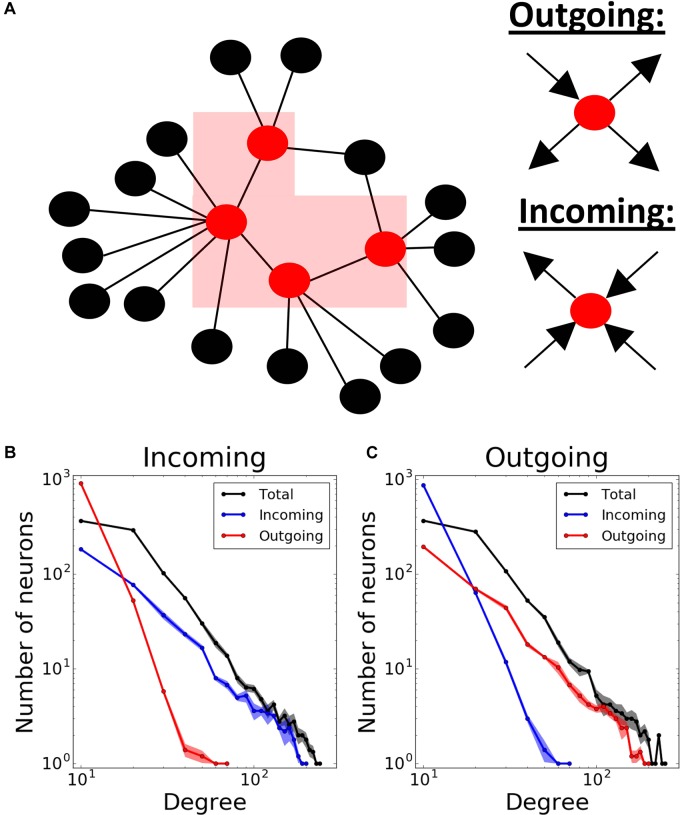
Modeling different scale-free network structures depending on the directionality at the hubs. **(A)** Scale- free networks are defined as “outgoing,” if the hubs have predominantly outgoing connections, and “incoming,” if the hubs have predominantly incoming connections. Total, incoming and outgoing degrees in both **(B)** incoming and **(C)** outgoing networks exhibit power-law distributions. Degree distributions are averaged over 5 different network realizations.

We used Barabasi – Albert algorithm (Barabasi et al., 1999) on a population of 1000 neurons to create a scale-free connectivity. We started with an all-to-all connected network of n neurons, and then expanded the network continuously by connecting new neurons to n pre-existing ones using preferential attachment principle: neurons with more connections have a higher chance to receive new connections. This results in a bidirectionally connected network with $\frac{n}{10}\%$ connectivity. Unless otherwise stated, we used *n* = 16 (1.6% connectivity) in our simulations. Then, we proceeded to make the connections unidirectional and defined two network transmission directions: incoming and outgoing. For that purpose, we first enumerated the neurons 1 to 1000 based on the time step they are added to the network. The earlier the neurons were added to the network, the higher chance they had to get new connections. Therefore, the neurons being assigned smaller numbers would eventually be more likely to have more connections. Then, we defined two different network structures according to the predominant directions of the connections at the hubs, i.e., nodes having a lot of connections to many other nodes (Boccaletti et al., 2006). We defined incoming networks as networks with hubs having majority of incoming connections. Therefore, we changed all bidirectional connections of the network into unidirectional connections from bigger to smaller-numbered neurons. Conversely, in outgoing networks, the hubs are dominated by outgoing connections. Therefore, the connections were directed from the neurons with smaller numbers to the ones with bigger numbers. Below, we will refer to these connectivity structures as “incoming” and “outgoing” networks, respectively (Figure 1A).

Finally, to obtain feedback connectivity, we randomly chose m% of all connections to change their directions. We defined this proportion (m%) as “direction ratio” in the paper. As a result, each neuron has $\frac{100}{m}-1$ times more incoming than outgoing connections on average in incoming networks, and vice versa in outgoing networks. Unless otherwise stated, we used 17% direction ratio in both network structures in our simulations. Consequently, the resulting networks have power-law degree distribution for their total, incoming and outgoing connections (Figure 1B,C).

We used integrate-and-fire excitatory neuron model to describe dynamics of each node. The current-balance equation of this neuron model for i-th neuron is

(1) $$\frac{\partial V_{i}\left(t\right)}{\partial t}=-\alpha V_{i}\left(t\right)+\gamma \underset{j}{\sum }J_{ij}S_{j}\left(t,t_{s}\right)+\beta I_{rand},$$

where *V_i_*(*t*) is the membrane potential of the i-th neuron, *J* denotes the adjacency matrix, γ = 0.25*V/s* is the synaptic strength, α = 0.3*ms*^-1^ is the inverse of the passive membrane time constant. The *I_rand_* is a random term, which is a 0.1 ms-wide rectangular current with an amplitude of 1, perturbing the neuronal dynamics with 100 Hz frequency; β = 6*V/s* is a term to modify the amplitude.

A neuron spikes when its membrane potential reaches *V_i_*(*t*) = 1. At the time of the spike, the voltage of the spiking neuron is reset to 0, and the neuron enters the refractory period of 5 ms (*t_ref_*). During this period, the neuron cannot receive any signals from its presynaptic connections (Burkitt, 2006).

There are no delays in the synaptic transmission. The postsynaptic signal arriving at each neuron is described by a double-exponential

(2) $$S_{i}\left(t,t_{s}\right)=e^{\frac{-\left(t-t_{s}{}_{{}_{i}}\right)}{T_{decay}}}-e^{\frac{-\left(t-t_{s_{i}}\right)}{T_{rise}}},$$

where *t_s_* is the last spike time of the i-th presynaptic excitatory neuron, *T_rise_* = 0.3*ms* and *T_decay_* = 3*ms* are rise and decay time constants, respectively.

For one set of our simulations (Figure 11), we added a population of inhibitory neurons consisting of 1000 neurons to the network. This population is randomly and unidirectionally wired with the same mean connectivity as excitatory population (1.6%). Moreover, inhibitory population sends connections to the excitatory one with 1.6% random connectivity, and vice versa. All parameters governing dynamics of inhibitory neurons are the same, except the sign in signal *S_i_*:

(3) $$\;S_{i}\left(t,t_{s}\right)=-\left(e^{\frac{-\left(t-t_{s_{i}}\right)}{T_{decay}}}-e^{\frac{-\left(t-t_{s_{i}}\right)}{T_{rise}}}\right),$$

### Implementation of Synaptic Transmission Failure

We defined a parameter, transmission probability *p_trans_* that provides a probability of a synapse passing (or failing to transmit) the signal, i.e., each synapse independently can pass (or fail) a presynaptic spike to a postsynaptic neuron. Here, we studied two realizations of this process: (1) activity-independent one, where transmission probability is constant (and the same for every synapse), and (2) activity-dependent one, where the probability of the synapse to succeed or fail depends on the time elapsed from the last spike of the postsynaptic neuron:

(4) $$p_{trans}\left(t\right)=1-p_{syn}\times e^{\frac{-\left(t-t_{last}-t_{ref}\right)}{T}},$$

where *p_syn_* is the base failure probability, *T* is the failure recovery time constant and *t_last_* is the last spike time of that neuron. Therefore, the term *t-t_last_-t_ref_* denotes for the time passed after the last spike time and its corresponding refractory period.

### Measures and Statistics

For all realizations of the network and its dynamics, we measured the MPC (mean phase coherence) between the neurons and the degree of the synchrony. The first measure allows us to assess the stability of the spatio-temporal pattern irrespective whether it is synchronous or asynchronous. Briefly, the instantaneous phase between two neurons is defined as

(5) $$\;\phi_{k}=2\pi \left(\frac{t_{2,k}-t_{1,k}}{t_{1,k+1}-t_{1,k}}\right),$$

where *t*_1, *k*_ is the time of the last spike of the neuron 1 before that of the neuron 2 (*t_2, k_*) and *t*_1, *k*+1_ is the time of the first spike of the neuron 1 after *t*_2, *k*_. Then the MPC between two neurons σ_1,2_ is

(6) $$\sigma_{1,2}=|\frac{1}{N}\overset{N}{\underset{k=1}{\sum }}e^{i\phi_{k}}|,$$

where *N* is the number of spike combinations at the two cells. The network measure of MPC, 〈σ〉, is the average of all pairs (Mormann et al., 2000).

The second measure indicates to what extent the neurons form synchronized pattern of activity. Here, the measure we used depends on the time-averaged fluctuations of the global voltage (σ_*V*_) over an extended period of time, normalized to the average of *N* individual neurons’ time-averaged fluctuations:

(7) $$\lambda =\sqrt{\frac{{\left[\sigma_{v}\right]}^{2}}{\frac{1}{N}\sum_{i=1}^{N}{\left[\sigma_{v_{i}}\right]}^{2}}}.$$

It is in the range of (0,1), increasing with synchronous activity (Golomb and Rinzel, 1993). The simulations were repeated 5 times, we calculated mean and its standard error to establish significance of the obtained results.

## Results

We used scale-free network structures, which are thought to represent functional network connectivity in the brain (Bullmore and Sporns, 2009). Scale-free connectivity provides a power-law distribution of nodal degrees resulting in a heterogeneous population of interconnected cells (Barabasi et al., 1999). We further differentiated network types by establishing hub directionality, in the sense that the highly interconnected cells (the hubs) may predominantly receive inputs from other parts of the network, or send outputs to other cells (Figure 1A). The example statistics of the connectivity for both of these cases are provided on Figure 1B,C, where the direction ratio is being established at 17%.

**FIGURE 2:**
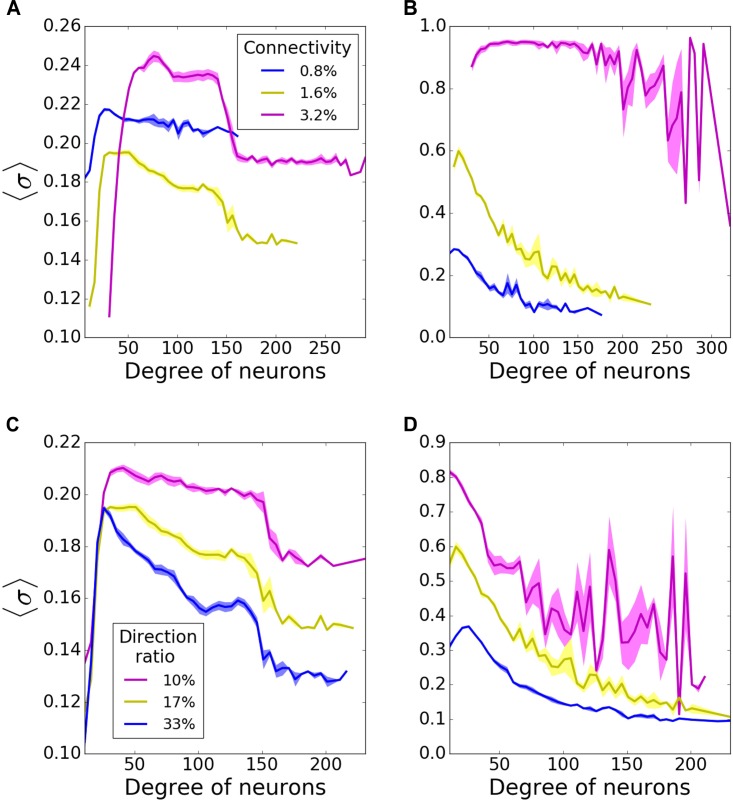
Nodal contribution to network-wide mean phase coherence (MPC) as a function of its degree for incoming **(A,C)** and outgoing **(B,D)** networks for different connectivities **(A,B)**, and direction ratios at the hubs **(C,D)**. In incoming networks, **(A)** increasing connectivity causes a bigger gap between coherences of high- and moderate-degree neurons, whereas **(C)** increasing direction ratio decreases MPC of all degrees. In outgoing networks, MPC of all degrees increases with **(B)** increasing connectivity and **(D)** decreasing direction ratio. MPCs are averaged over 5 degrees and results are averaged over 5 randomized network realizations.

First, to establish a baseline, we investigated pattern formation in the networks without failure, as a function of the mean connectivity (Figure 2A,B) and direction ratio (Figure 2C,D) in both incoming (Figure 2A,C), and outgoing (Figure 2B,D) networks. In incoming networks, the histograms of average MPC (〈σ〉) as a function of neuron degrees, suggest that low degree neurons always have relatively lower MPC than the rest of the network, regardless of the connectivity and direction ratio of the network, because of the lack of common input they get. However, this difference is more pronounced for higher connectivities (Figure 2A). Generally, we observe that for low connectivity the network has relatively few connections and thus it remains less heterogeneous in terms of nodal degree. As the connectivity is increased, two competing mechanisms emerge – the networks become more heterogeneous, but at the same time stronger connectivity leads to more synchronous dynamics, as is commonly observed. However, even though nodal contributions exhibit different patterns for different connectivities, these differences are only minimal in incoming networks.

Connectivity has a bigger impact on outgoing networks (Figure 2B). Higher connectivities result in more significant increases in MPC for all degrees. That is not surprising as hubs are the synchronizing agent to the rest of the network when they drive network activity. Unlike incoming networks, outgoing ones have the highest MPC for the neurons with lowest degrees, for 0.8, and 1.6% connectivities. The reason is that in these type of networks, neurons with lowest degrees receive signals from hubs and form synchronized clusters. This trend disappears for 3.2% connectivity though, since the network is saturated and neurons with all degrees are coherent. The bigger fluctuations for highest degrees at 3.2% connectivity might be due to the lower input they get from the network.

We then investigated how direction ratio (as defined in methods) affects network coherence. In incoming networks, MPC for all degrees is increased overall for lower direction ratios (more incoming connections at the hubs), although the change is not very significant (Figure 2C). However; there is a more substantial rise in the case of outgoing networks, when hubs send more outgoing connections (Figure 2D). These results point in the direction that the overall synchrony of the network is strongly dependent on the number of outgoing connections emanating from the hubs, rather than incoming ones. For the rest of our simulations, unless stated otherwise, we decided to use incoming and outgoing network structures with 1.6% connectivity, 17% direction ratio.

Finally, we varied the frequency of random external kick *I_rand_*. In case all neurons are disconnected (*p_trans_* = 0.0), spike frequency increases with increasing *I_rand_* frequency, as expected. At the same time, as expected, the MPC decreases with more frequent *I_rand_*. When all the connections are present (*p_trans_* = 1.0), both spike frequencies and MPCs are only minimally increased for higher frequencies of *I_rand_*, since network connections dominate pattern formation. For the rest of our simulations, we chose the frequency as 100 Hz. This value results in a spike frequency lower than 200 Hz, the maximum frequency the network can fire due to the 5 ms refractory period, when *p_trans_* = 1.0. Also, it introduces enough randomness to the network to make them spike less coherently when *p_trans_* = 0.0. These results are briefly summarized in Supplementary Figure S1.

### Activity – Independent Synaptic Failure

**FIGURE 3:**
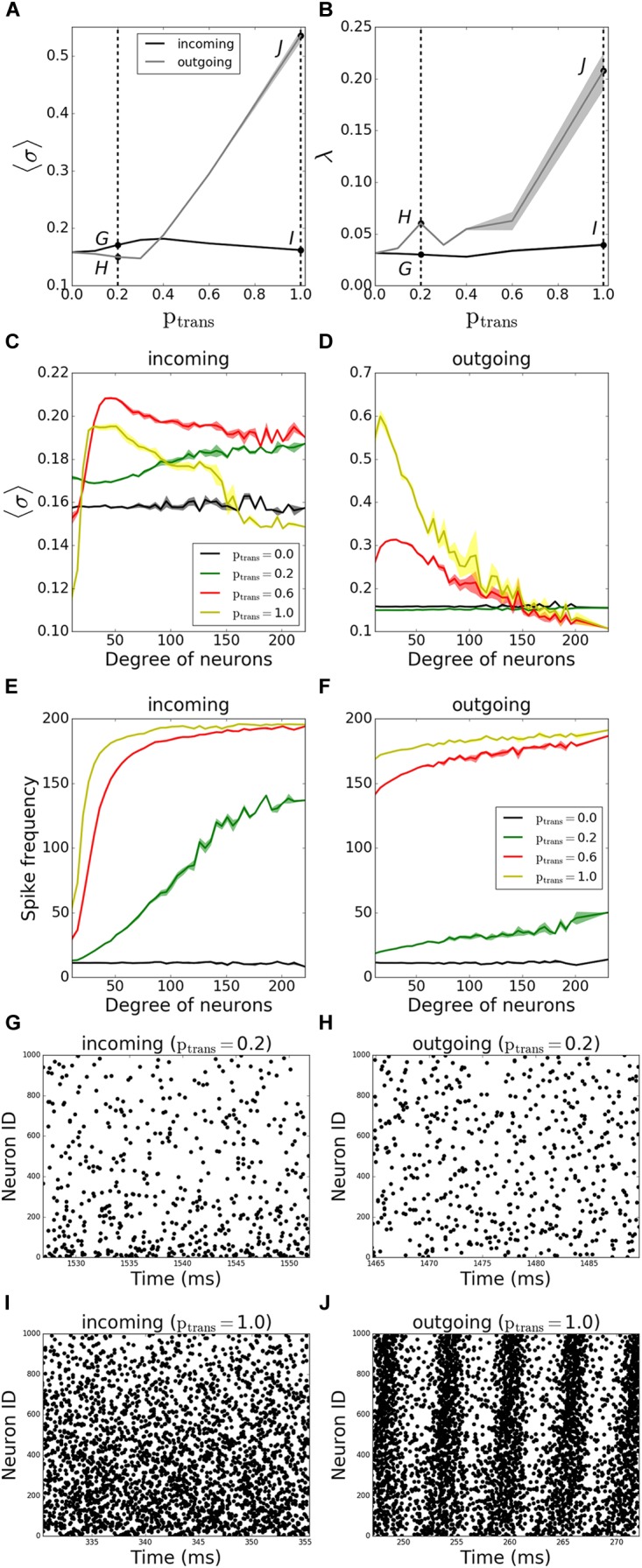
Pattern formation in the networks with activity-independent synaptic failure shows that outgoing networks become more coherent and synchronous due to a more uniform spike frequency distribution throughout the network. Signals are transmitted through synapses of a neuronal network with a constant probability *p_trans_*. **(A)** MPC and **(B)** synchrony measure for incoming (black line) and outgoing (gray line) networks. **(C,D)** Participation in pattern formation as a function of nodal degree for activity-independent transmission failure. Histograms of MPC as a function of degree of neurons for incoming **(C)** and outgoing **(D)** networks, averaged over 5 degrees. **(E,F)** Spike frequencies of different degrees in incoming **(E)** and outgoing **(F)** networks, averaged over 5 degrees. Raster plots for incoming **(G,I)** and outgoing **(H,J)** networks for parameter values indicated on panels **(A)** and **(B)** [Points *G–J* correspond to panels **(G–J)**]. Lower neuron ID means higher degrees and vice versa (see Figure 12A,E for degrees corresponding to neuron IDs). Results are averaged over 5 simulations.

We first investigated the history-independent transmission probability, where *p_trans_* is constant. We compared the pattern formation (i.e., the MPC and synchrony) for the outgoing and incoming networks as we gradually varied *p_trans_* between 0 and 1 for both network types. We observed that outgoing networks are more sensitive to synaptic failure than incoming ones, as they become more coherent and synchronous with increasing synaptic transmission (Figure 3A,B).

We then investigated how the MPC and synchrony forms within the network as a function of degree number of constituent neurons. The histogram of the average MPC constructed as a function of connection number for varying degrees of incoming networks (Figure 3C) suggests that, for full transmission (*p_trans_* = 1.0), moderate-degree neurons of incoming networks have higher average MPC values than low-degree or high-degree neurons. This seems intuitive as the neurons with very few connections don’t get enough input to form stable patterns, whereas a few cells with a large number of inputs cannot synchronize with the rest of the population, as their frequency is significantly different due to widely varying number of excitatory inputs (Figure 3E). This trend is reversed for higher failure rate (*p_trans_* = 0.2), with hubs being more coherent than the rest of the network.

Moreover, for *p_trans_* = 0.6, neurons fire more coherently than when *p_trans_* = 1.0 for all degrees. This provides evidence that failure can promote more coherent behavior, as the input received by different degrees becomes more uniform with failure.

The same histogram for outgoing networks (Figure 3D) shows that, for the same *p_trans_*, the average MPC values are higher than the incoming case. This is due to a more balanced input levels across the neurons in the network, i.e., a more balanced frequency distribution throughout different degrees (Figure 3F). In general, higher-degree neurons have lower average MPC in the outgoing case, and this effect is the most pronounced for higher values of *p_trans_*. The example raster plots of the observed dynamics are presented on Figure 3G–J, with the corresponding values of *p_trans_* marked on Figure 3A,B.

To assess better the specific role of neurons having different degree numbers (i.e., number of connections) on pattern formation, we divided the neurons in each network into 3 groups depending on their total degree (i.e., the sum of their incoming and outgoing connections). The groups were formed so that the total number of the connections in each group is equal. Thus, the number of neurons in each group is inversely proportional to the average degree of individual neurons in the groups, resulting in equal number of connections per group; neurons with degrees less than 24 are in “Group 1,” between 24 and 48 degrees are in “Group 2”, and with more than 48 degrees are in “Group 3.” In terms of number of neurons, the groups consist on average of 533, 342, 125 neurons, respectively. The signals coming through the incoming connections to a given group are tested against different transmission probabilities *p_trans_*, while the rest of the connections don’t fail at all (*p_trans_* = 1.0), to see the individual effects of the failure of signals coming to different degrees on overall pattern formation.

**FIGURE 4:**
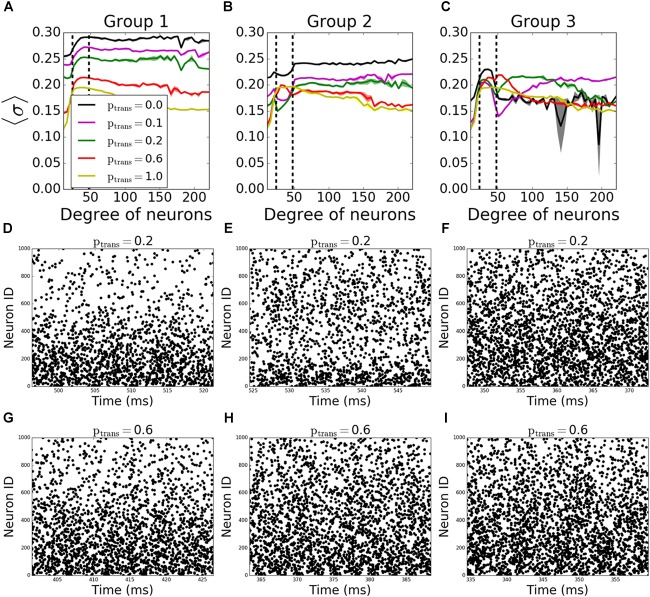
Nodal contribution to network-wide MPC as a function of its degree for **incoming networks** show an increased coherence of hubs, when they fail to receive signals. Neurons in the network are grouped according to their degrees. Groups are assigned such that neurons in each group has equal total number of connections. Neurons with degrees 1–23 constitute group 1 [left of the first dashed line in panels **(A–C)**], neurons with degrees 24–47 constitute group 2 (between dashed lines), and neurons with degrees >48 constitute group 3 (right of the second dashed line). Signals are transmitted to group 1 **(A)**, group 2 **(B)**, and group 3 **(C)** with the probability *p_trans_*, while the rest of the network receives signals without failure. Raster plots for the cases in **(A–C)** are shown in **(D–G)**, **(E–H)**, and **(F–I)**, respectively, for two values of *p_trans_*. Lower neuron ID means higher degrees and vice versa (see Figure 12A,E for degrees corresponding to neuron IDs).

In incoming networks, the response of MPC to the network manipulations is generally small. Interestingly we observe that the failing signals coming to Group 1 (Figure 4A, example raster plots on Figure 4D,G) and Group2 (Figure 4B, example raster plots on Figure 4E,H) result in an overall increase in MPC values of the unaffected groups, with Group 1 having a bigger effect than Group 2. The reason is that preventing lower degree groups from receiving signals make them fire only as a result of *I_rand_*, decreasing their overall firing frequency as the synapses fail. Lowering the frequency in these two groups reduced the frequency in all other groups leading to observable increase of coherence.

The progressive failure of incoming connections to Group 3 has a more complicated effect. We observe a higher coherence of that group than the rest of the network for 0.0 < *p_trans_* < 1.0 (Figure 4C, example raster plots on Figure 4F,I). This increase as a function of reduction of the transmission probability in the hub group brings the magnitude of the incoming signal to the hub cells to be similar to that of the intermediate group, increasing effectively the coherent backbone of the network. For *p_trans_* = 0.1, we observe that the MPC of hubs are equal and higher than no failure case (*p_trans_* = 1.0), and this equalizing effect disappears with increasing *p_trans_*. This effect is further confirmed through observation which neurons from Group 3 show increased synchronization as a function of increased failure – for lower transmission rates the neurons within that group with higher degrees exhibit increased coherence, whereas for higher transmission, the cells with lower degrees show increase of coherence.

**FIGURE 5:**
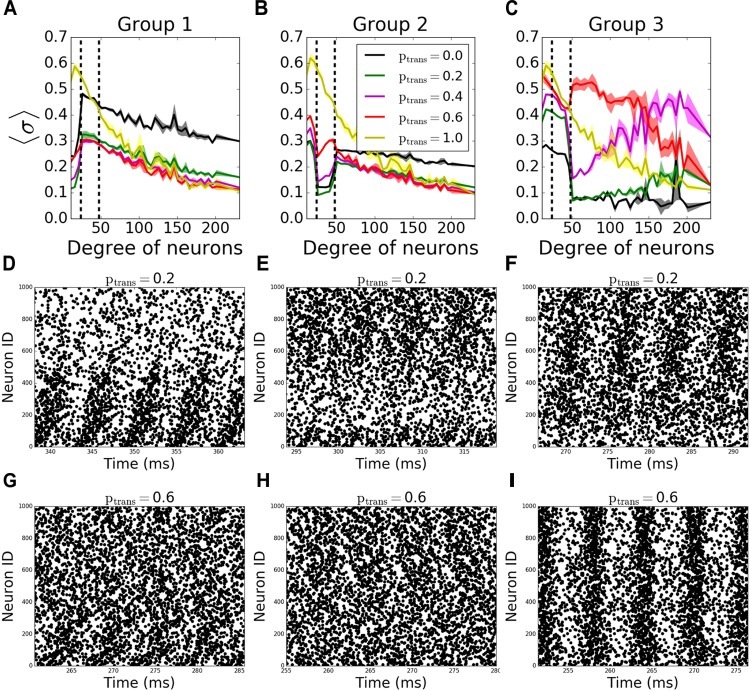
Nodal contribution to network-wide MPC as a function of its degree for **outgoing** **networks** show an increased coherence of hubs for *p_trans_* ≥ 0.4 than when there’s no failure. Neurons in the network are grouped according to their degree. Groups are assigned such that neurons in each group has equal total number of connections. Neurons with degrees 1–23 constitute group 1 [left of first dashed line in panels **(A–C)**], neurons with degrees 24–47 constitute group 2 (between dashed lines), and neurons with degrees >48 constitute group 3 (right of second dashed line). Signals are transmitted to group 1 **(A)**, group 2 **(B)**, and group 3 **(C)** with the probability *p_trans_*, while the rest of the network receive signals without failure. Raster plots for the cases in **(A–C)** are shown in **(D–G)**, **(E–H)**, and **(F–I)**, respectively, for two values of *p_trans_*. Lower neuron ID corresponds to cells with higher degrees (see Figure 12A,E for degrees corresponding to neuron IDs).

In outgoing networks, even moderate increase in failure of Group 1 decreases Group 1 and Group 2’s MPC significantly, but hubs (Group 3) are not affected. When *p_trans_* = 0.0, we observe overall decrease in frequency which leads to increase in reported coherence (Figure 5A). The same holds for the case when Group 2 fails to receive signals (Figure 5B). However, when Group 3 is disconnected, the same reversal effect is observed as in incoming case (Figure 5C), but with significantly higher observed changes in MPC. This is again due to the homogenization of the received signals by neurons having different degree. As before, Figure 5D–I show example raster plots for two transmission values: *p_trans_* = 0.2 and *p_trans_* = 1.0.

### Activity – Dependent Case

The second case we studied is when the transmission probability depends on the spiking history of the postsynaptic neurons, i.e., the signal coming to the postsynaptic neuron, which more recently fired, has a higher chance to fail due to the postsynaptic receptor sensitivity. This case may be biologically more relevant, since it is known that neurodegenerative diseases, such as AD and Parkinson’s, have lower levels of postsynaptic ionotropic receptors (Dinamarca et al., 2012; Xu et al., 2012). As a result, this may cause a more effective desensitization of the neurotransmitter-gated ion channels in case of higher frequency stimulation via spiking presynaptic neurons (Rosenmund and Mansour, 2002; Papke et al., 2011). Moreover, higher activity is shown to result in regional vulnerability to amyloid-β deposition in AD, which causes synaptic failure (Bero et al., 2011).

In this case, we vary two parameters; the base failure probability *p_syn_* and failure recovery time constant *T*. Here, *p_syn_* = 1 indicates the possibility of complete failure of the synapse. We vary *T* between 0 ms and 5000 ms, with *p_syn_* = 1 and *T* = 5000*ms* being a disconnected network.

**FIGURE 6:**
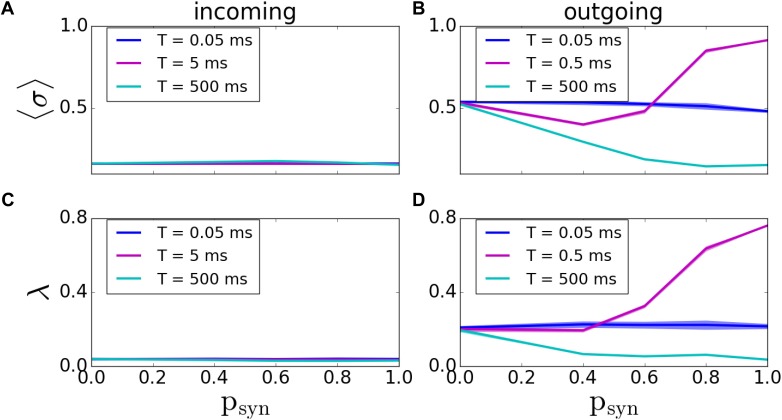
Pattern formation in the incoming and outgoing networks with activity-dependent synaptic failure. **(A,B)** MPC and **(C,D)** synchrony for incoming **(A,C)**, and outgoing **(B,D)** networks as a function of *p_syn_* for three different values of failure recovery time constants *T*. There is a dramatic increase in MPC and synchrony with increasing synaptic failure (*p_syn_*) for a moderate *T* in outgoing networks.

As before, we first assessed the overall degree of pattern formation in both types of networks. In incoming networks, we didn’t see any significant changes in MPC and synchrony for various *T* and *p_syn_* values (Figure 6A,C). However; for outgoing networks, we observed an overall decrease in the network coherence for increasing *p_syn_*. For fast synaptic recovery, this decrease is significantly smaller (Figure 6B,D). Interestingly, however, for *T* = 0.5*ms*, we observed a dramatic increase of both MPC and synchrony as *p_syn_* tends to unity.

**FIGURE 7:**
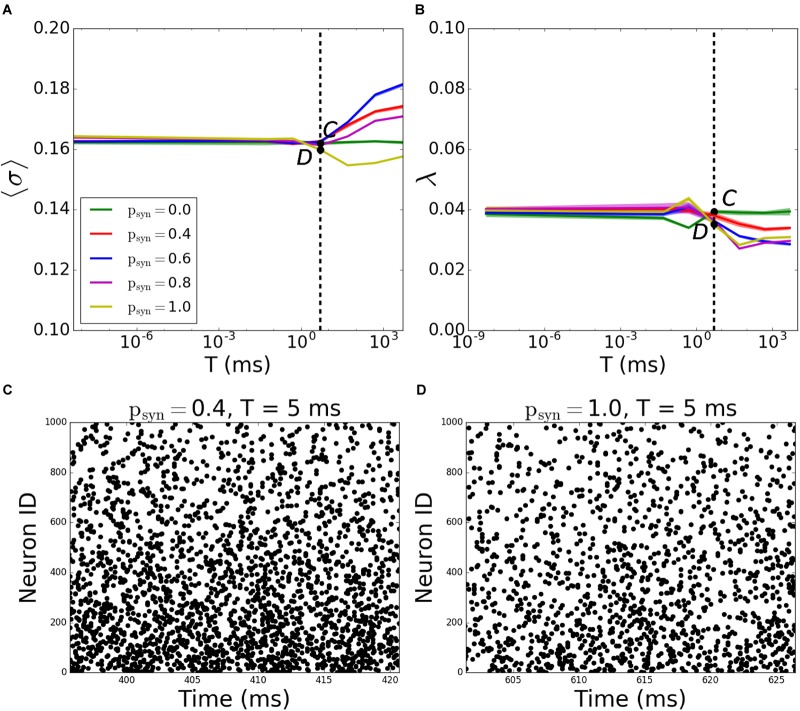
Pattern formation in the incoming networks with activity-dependent synaptic failure as a function of failure recovery time constant *T*. **(A)** MPC and **(B)** synchrony as a function of time constant *T* for different values of *p_syn_*. **(C,D)** Raster plots depicting network activity for parameter values marked on **(A,B)** [Points *C* and *D* correspond to panels **(C,D)**]. The specific parameter values of *T* and *p_syn_* are listed on top of each raster plot. Lower neuron ID corresponds to cells with higher degrees (see Figure 12A,E for degrees corresponding to neuron IDs).

A more systematic scan of time constants *T* reveals that for incoming networks, the network starts getting disconnected for *T* > 5*ms*. When *T* = 5000*ms*, the MPC and synchrony values are the same as activity-independent case, meaning that *T* is large enough that *p_trans_* ≈ 1 - *p_syn_* (Figure 7A,B; the corresponding raster plots are displayed on Figure 7C,D).

**FIGURE 8:**
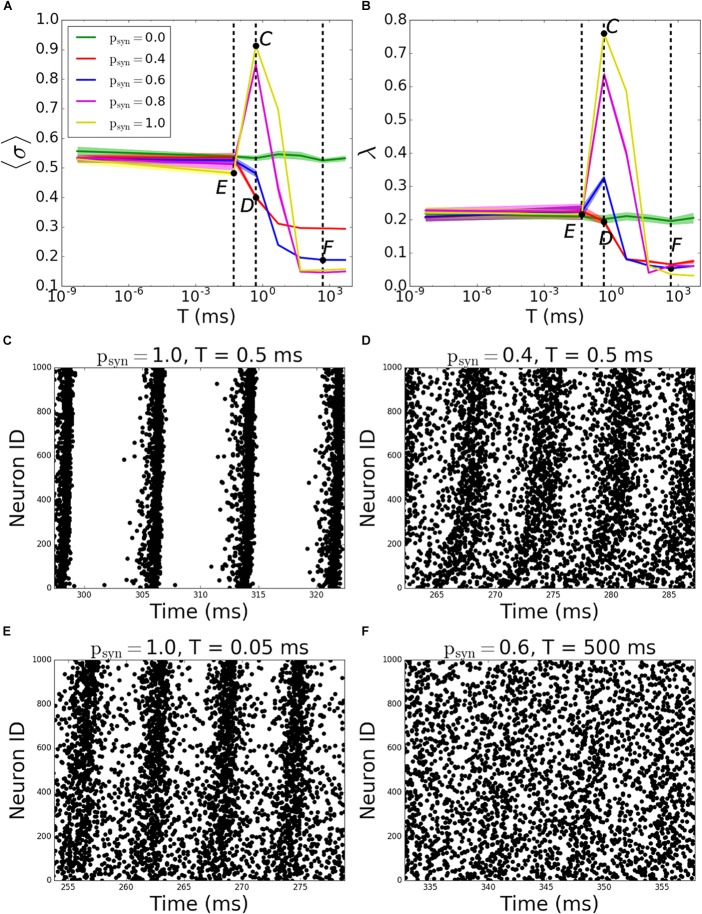
Pattern formation in the outgoing networks with activity-dependent synaptic failure as a function of failure recovery time constant *T*. **(A)** MPC and **(B)** synchrony as a function of time constant *T* for different values of *p_syn_*. **(C–F)** Raster plots depicting network activity for parameter values marked on **(A,B)** [Points *C–F* correspond to panels **(C–F)**]. The specific parameter values of *T* and *p_syn_* are listed on top of each raster plot. Lower neuron ID corresponds to cells with higher degrees.

The behavior of outgoing networks is similar to the one described above except for *T* = 0.5*ms*, where we observed a large peak in both MPC and synchrony, as *p_syn_* tends to one (Figure 8A,B; the corresponding raster plots are displayed on Figure 8C–F).

**FIGURE 9:**
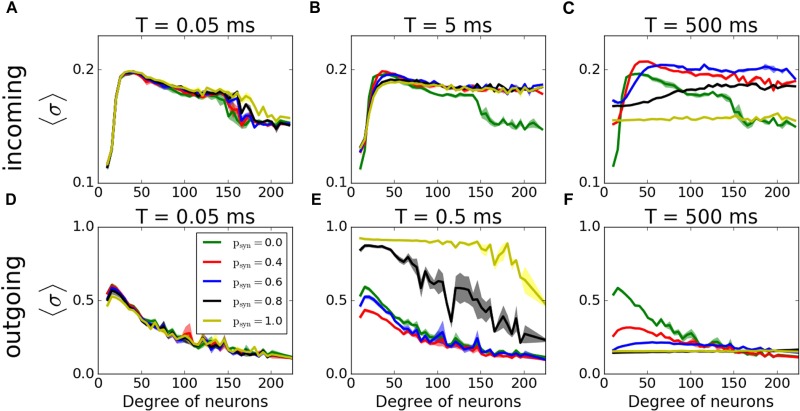
Histograms of MPC as a function of degree of neurons of incoming **(A–C)** and outgoing **(D–F)** networks for varying failure recovery time constants *T* (denoted on top of each panel). Participation in pattern formation as a function of nodal degree for activity-dependent transmission failure shows an increased coherence with increased failure for moderate *T* in both networks.

We then have investigated how synaptic failure interacts with neurons with specific nodal degree to form activity pattern within the network (Figure 9). For incoming networks and for large *T* (Figure 9A–C) the degree dependence is largely similar to that of constant *p_trans_*, described in the previous section. The group with the largest coherence is the group having intermediate degree values. For small *T*, as expected, *p_syn_* does not influence the overall coherence levels as the transmission probability rapidly recovers and *p_trans_* ≈ 1.0. For larger time constants, the overall level of coherence depends on the *p_syn_*, as in the case of activity-independent case (Figure 3C). For a moderate time constant (*T = 5ms*), however, we observe that hubs have higher coherence when *p_syn_*≠0.0 than when *p_syn_* = 0.0, which means that failure of spikes results in a more coherent behavior of hubs, even though globally there’s no significant change in the network’s MPC. This is driven by synaptic failure capacity to equalize input to the cells across the network.

**FIGURE 10:**
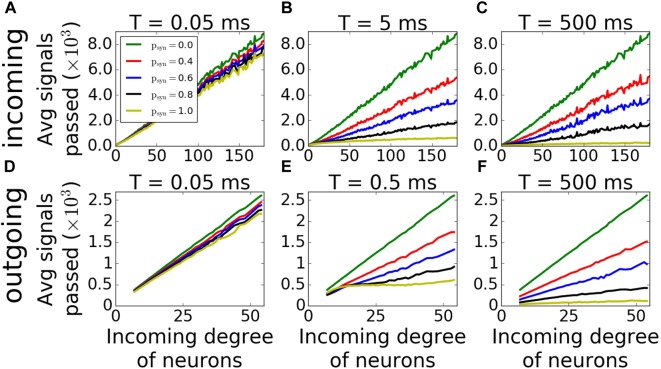
Average input magnitude to a neuron as a function of nodal degree for activity-dependent synaptic failure. Histograms of average number of signals transmitted as a function of incoming degree of neurons of incoming **(A–C)** and outgoing **(D–F)** networks for varying failure recovery time constants *T* (denoted on top of each panel) show that for *T* = 0.5*ms* and *p_syn_* = 1.0, outgoing networks receive the same amount of signals, and independent from their degrees.

For outgoing networks, generally the same is true for low and high values of *T* (Figure 9D,F) as in incoming case. However, for the value *T* = 0.5*ms* (Figure 9E), we observe a complete reversal of the overall network coherence, with the largest coherence happening for the largest *p_syn_* and approaching to one. The MPC is then largely independent of the neuronal degrees, except the highest ones, where MPC starts dropping. To understand the reason behind this sudden increase, we measured the average number of signals transmitted to each neuron as a function of its incoming degree. The histograms (Figure 10) suggest, that for low *T*, for both incoming and outgoing networks, there is a linear proportionality between the input and the incoming degree number (Figure 10A,D). For larger values of *T*, the signal curves depend directly on the value of *p_syn_* and for large *p_syn_* they saturate for large degree values (Figure 10B,C,E,F), making the amount of signal received by neurons largely independent of degree. However, only for outgoing networks and *T* = 0.5*ms*, all neurons, independent from their incoming degrees, receive the same number of the signals, which is significantly different from zero (Figure 10E). This suggest that at this specific *T* range, all cells in the network receive about the same input magnitude allowing them to synchronize across the entire system.

To see if similar results would be observed with different connectivities and direction ratios, we simulated various connectivity fractions and direction ratios for different time constants (*T* = 0.05, 5, 500*ms* for incoming networks and *T* = 0.05, 0.5, 500*ms* for outgoing networks). In incoming case, the reversal effect of MPC increase of hubs for higher *p_syn_* at a moderate time constant (*T = 5ms*) is not observed for lower or higher connectivities (Supplementary Figure S2). However, increasing direction ratio makes this effect more pronounced, since increasing outgoing connections at hubs makes the network more balanced overall (Supplementary Figure S3). In outgoing networks, this dramatic increase of overall coherence is still observed, and it is more pronounced for higher connectivities (Supplementary Figure S4) and lower direction ratios (Supplementary Figure S5), since the amount of outgoing connections from hubs is increased in both cases, resulting in a more coherent network overall.

**FIGURE 11:**
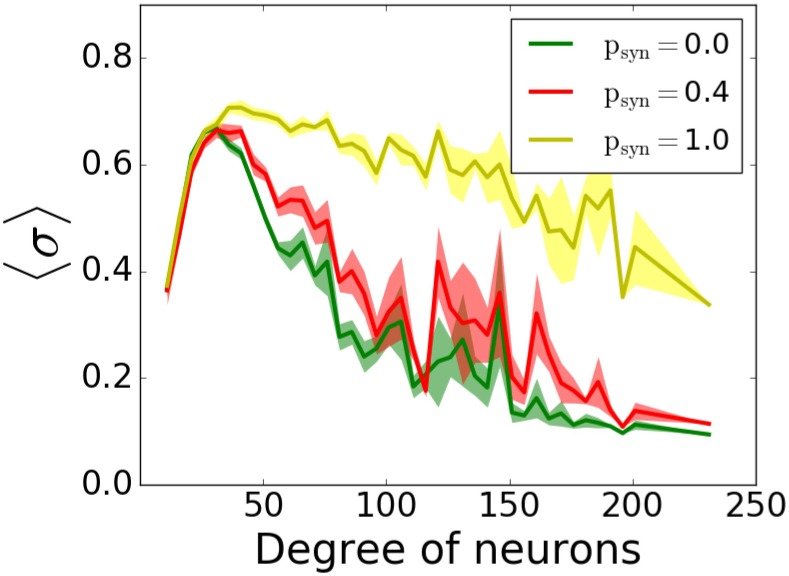
Participation in pattern formation as a function of nodal degree for activity-dependent transmission failure for mixed excitatory-inhibitory networks. Histograms of MPC as a function of degree of neurons for *T* = 0.5 ms and varying *p_syn_* levels show emergence of highly synchronous state for high values. The networks is composed of 1000 excitatory and 1000 inhibitory neurons. Excitatory neurons are wired as outgoing scale-free networks, while inhibitory neurons have random connections to both inhibitory and excitatory population with the same connectivity as the one within excitatory population (1.6%).

Lastly, we included inhibitory neurons to the network, since they are known to have significant effects on pattern formation in cortical networks (Vreeswijk and Sompolinsky, 1996). We simulated outgoing networks with an inhibitory population for *T* = 0.5*ms* and various *p_syn_*. Our results (Figure 11) suggest that there’s still that reversal effect as we’ve seen in Figure 9E, i.e., increasing *p_syn_* eventually increases the overall network coherence, when *p_syn_* approaches to 1.

## Discussion

**FIGURE 12:**
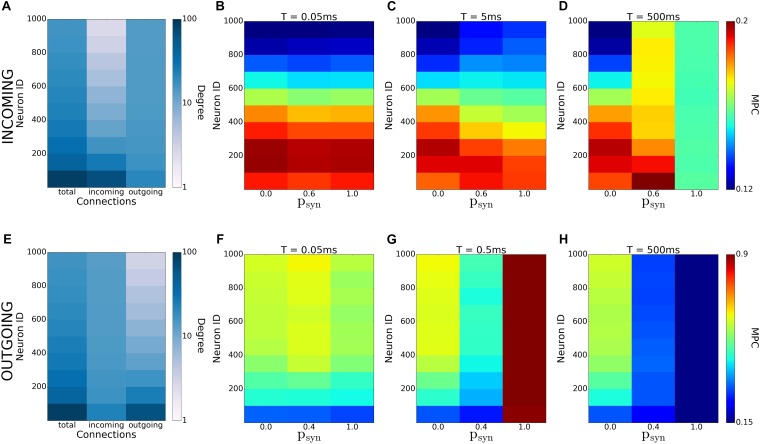
Emergence of coherence patterns in scale-free networks. Heatmaps of degrees **(A,E)** and MPC [**(B–D)** and **(F–H)**] for incoming **(A–D)**, and outgoing **(E–H)** networks show dependence of network-wide pattern formation on parameters of activity-dependent synaptic failure (*T* and *p_syn_*). Each color is an average of the values for 100 neurons.

In this study, we systematically analyzed how synaptic failure affects two complementary (incoming and outgoing) scale-free network structures. We studied the cases when synaptic transmission probability is activity-independent and activity-dependent. In the first case, we have found that targeted synaptic failure to neuronal population having different nodal degrees, has differential effects on pattern formation in the network. When synaptic failure was activity dependent, we observed that structural features of networks don’t map onto functional connectivity (Figure 12), but rather, synaptic failure may result in differential spatio-temporal patterning dependent on failure recovery time constant *T* and base failure probability *p_syn_*. Moreover, the two network structures, (incoming and outgoing) behave differently, with outgoing networks displaying overall a larger degree of coherence/synchrony and a higher dependence on transmission probability. This is especially evident for the activity-dependent transmission probability, where the outgoing networks exhibit an increased level of coherence for a large base failure probability (*p_syn_*) for a specific value of the failure recovery time constant (*T* = 0.5*ms*).

This abrupt increase in synchrony and coherence as a result of synaptic failure is unexpected and possibly paints a more complex picture of possible network interactions in the brain. It was hypothesized that anesthetics act predominantly on the network hubs and overall decrease the level of coherence across brain networks, leading to the loss of consciousness (Lee et al., 2013). Similarly, hubs are shown to be more vulnerable to amyloid deposition due to their high activity rate, causing the disruption of large-scale coherence in the brain and eventually AD (Buckner et al., 2009). In addition, numerical studies on scale-free networks suggest that they are robust against the random removal of nodes and the change in their synchronization process is insignificant in case 5% of their total nodes are randomly removed. However, when hubs are targeted, only the removal of 1% of the total nodes is enough to divide the network into subnetworks and to disrupt network synchronization (Albert et al., 2000; Jinhu and Guanrong, 2005; Boccaletti et al., 2006; Li et al., 2011). We, however, show that, depending on the network type, preferential deactivation of hubs and activity-dependent degeneration might lead to increased phase coherence and synchrony. Further investigation on human brain networks may be necessary to determine whether there’s an overall increased coherence phase before the decrease of large-scale coherence in such cases as application of anesthetics or AD. That may be a useful biomarker for AD as well as a significant contribution to explain impact on anesthetics on human brain.

## Author Contributions

MZ designed the research, oversaw the execution, and participated in writing the manuscript. MB executed the research and wrote the manuscript.

## Conflict of Interest Statement

The authors declare that the research was conducted in the absence of any commercial or financial relationships that could be construed as a potential conflict of interest.

## Supplementary Material

FIGURE S1 Network spike frequency **(A,C)** and mean phase coherence **(B,D)** for various frequencies of random input *I_rand_*, for incoming **(A,B)** and outgoing **(C,D)** networks. Results are averaged over 5 randomized network realizations.

FIGURE S2 Nodal contribution to network-wide MPC as a function of its degree for incoming networks for different connectivities and failure recovery time constant *T*. The increase in MPC of hubs with higher failure cannot be observed for lower or higher connectivities for *T* = 5 ms. MPCs are averaged over 5 degrees and results are averaged over 5 randomized network realizations.

FIGURE S3 Nodal contribution to network-wide MPC as a function of its degree for incoming networks for different direction ratios and failure recovery time constant *T*. Higher direction ratios result in a more obvious increase in MPC of hubs for *T* = 5 ms when there’s more failure. MPCs are averaged over 5 degrees and results are averaged over 5 randomized network realizations.

FIGURE S4 Nodal contribution to network-wide MPC as a function of its degree for outgoing networks for different connectivities and failure recovery time constant. Higher connectivities result in a bigger increase in MPC for *T*=0.5 ms with higher failure *p_syn_*. MPCs are averaged over 5 degrees and results are averaged over 5 randomized network realizations.

FIGURE S5 Nodal contribution to network-wide MPC as a function of its degree for outgoing networks for different direction ratios and failure recovery time constant *T*. For *T* = 0.5 ms, the increase in MPC values of *p_syn_* = 1.0 is more pronounced for lower direction ratios. MPCs are averaged over 5 degrees and results are averaged over 5 randomized network realizations.
