# Supplementary figures and images for: Synaptic Failure Differentially Affects Pattern Formation in Heterogenous Networks
Source: Front Neural Circuits. 2019 May 8;13:31. doi: 10.3389/fncir.2019.00031 (PMC6519395; doi:10.3389/fncir.2019.00031)

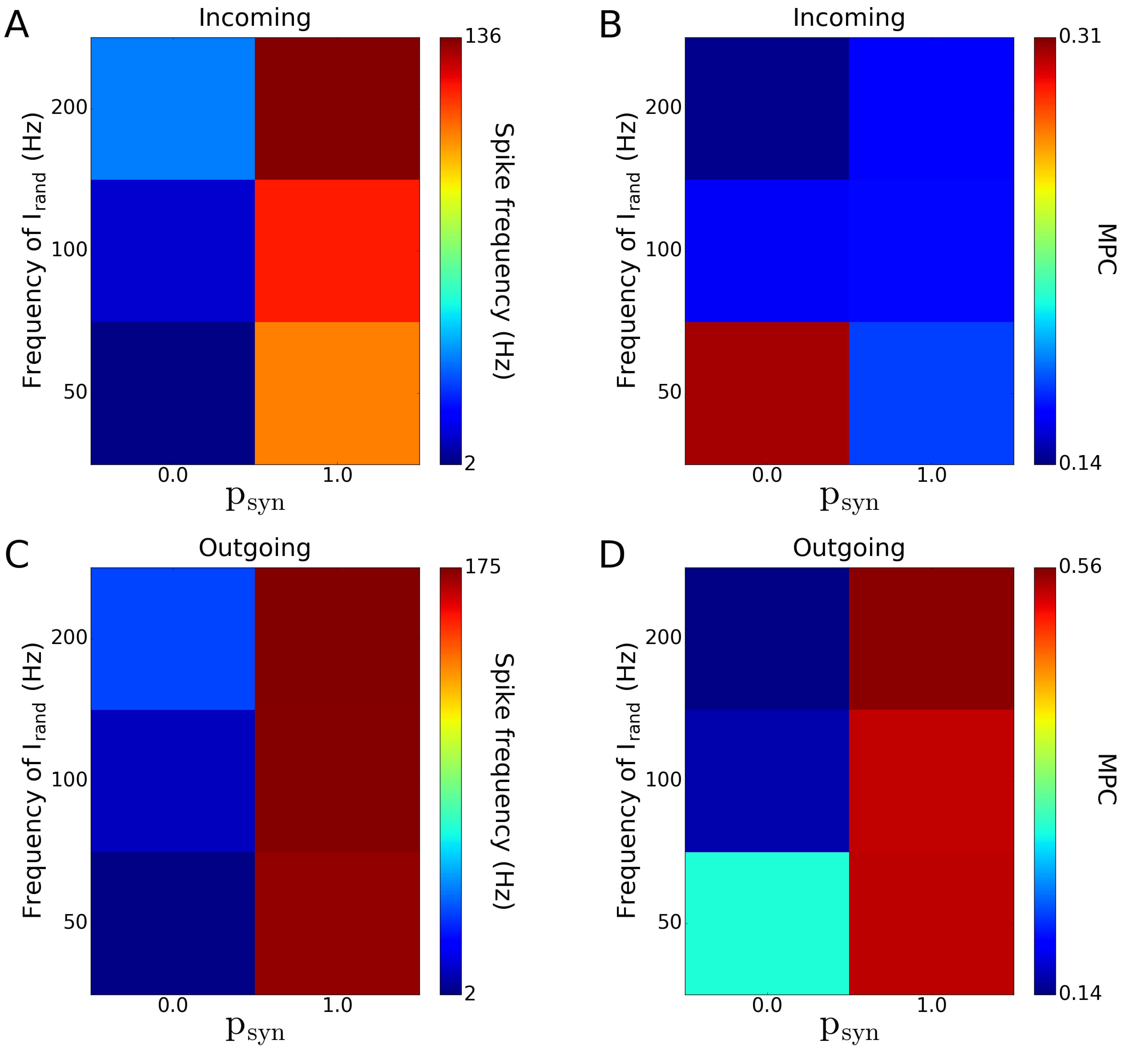

Supplement: FIGURE S1 — Network spike frequency (A,C) and mean phase coherence (B,D) for various frequencies of random input Irand, for incoming (A,B) and outgoing (C,D) networks. Results are averaged over 5 randomized network realizations. [file Image_1.JPEG]

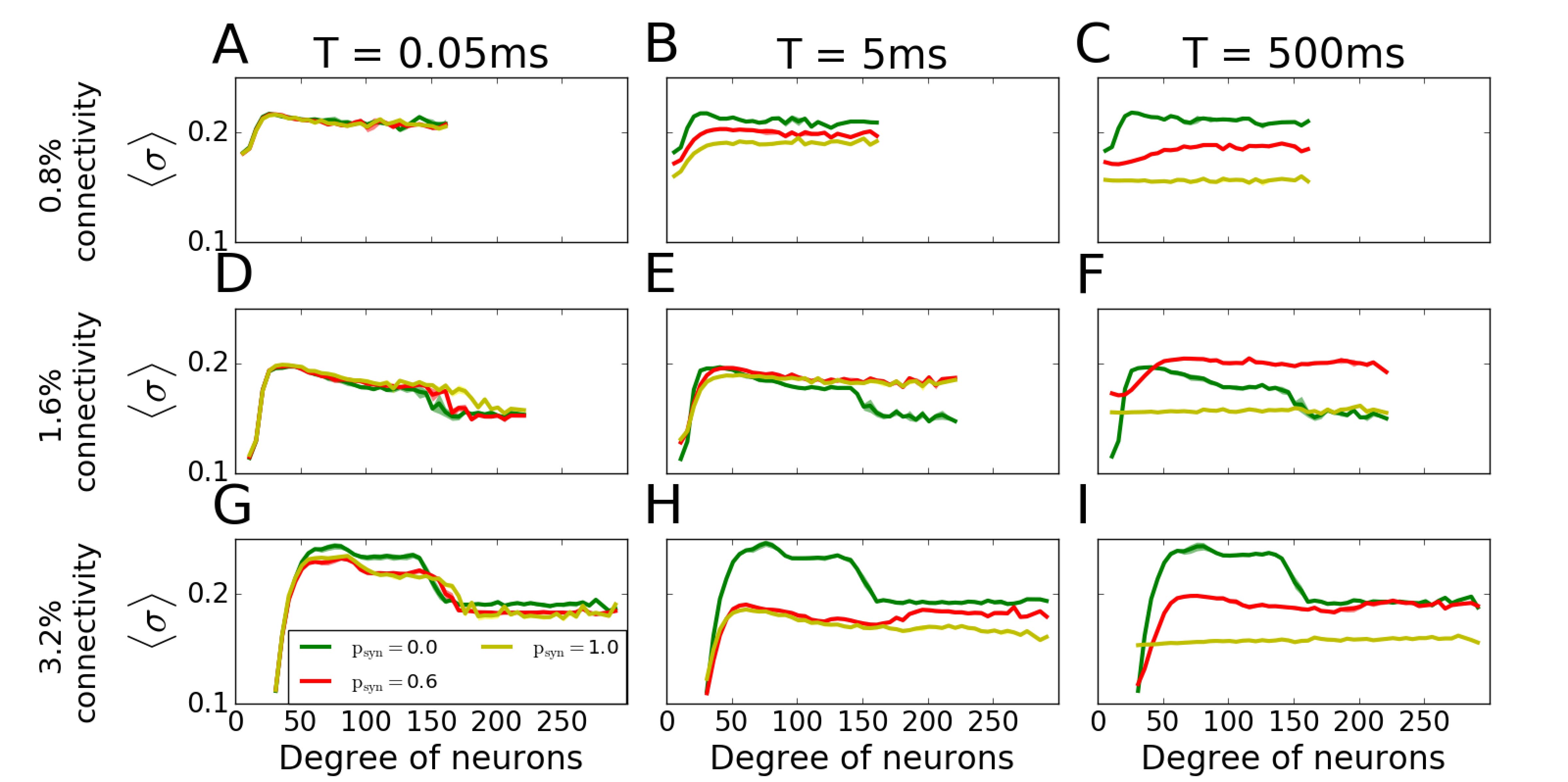

Supplement: FIGURE S2 — Nodal contribution to network-wide MPC as a function of its degree for incoming networks for different connectivities and failure recovery time constant T. The increase in MPC of hubs with higher failure cannot be observed for lower or higher connectivities for T = 5 ms. MPCs are averaged over 5 degrees and results are averaged over 5 randomized network realizations. [file Image_2.JPEG]

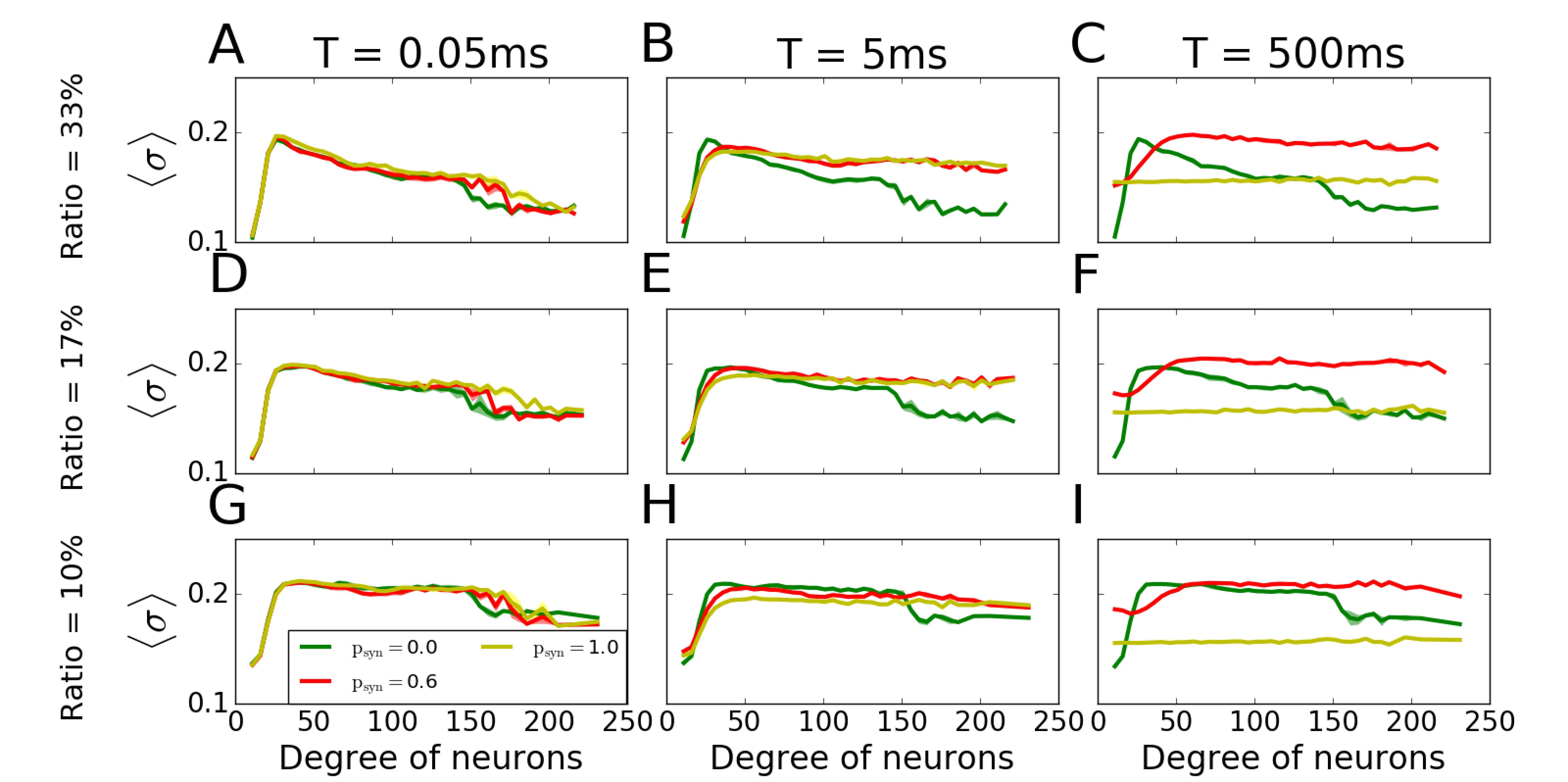

Supplement: FIGURE S3 — Nodal contribution to network-wide MPC as a function of its degree for incoming networks for different direction ratios and failure recovery time constant T. Higher direction ratios result in a more obvious increase in MPC of hubs for T = 5 ms when there’s more failure. MPCs are averaged over 5 degrees and results are averaged over 5 randomized network realizations. [file Image_3.JPEG]

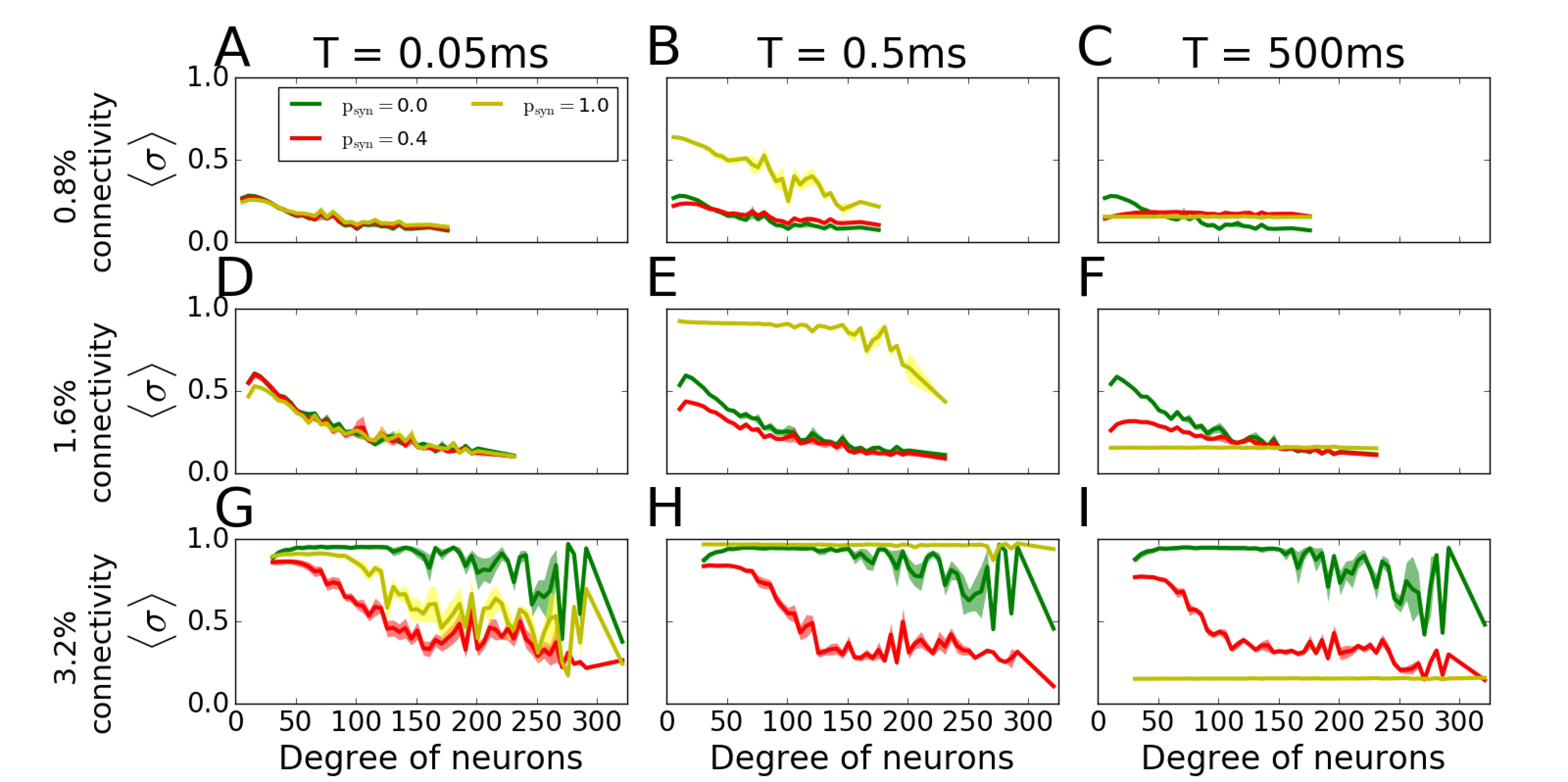

Supplement: FIGURE S4 — Nodal contribution to network-wide MPC as a function of its degree for outgoing networks for different connectivities and failure recovery time constant. Higher connectivities result in a bigger increase in MPC for T=0.5 ms with higher failure psyn. MPCs are averaged over 5 degrees and results are averaged over 5 randomized network realizations. [file Image_4.JPEG]

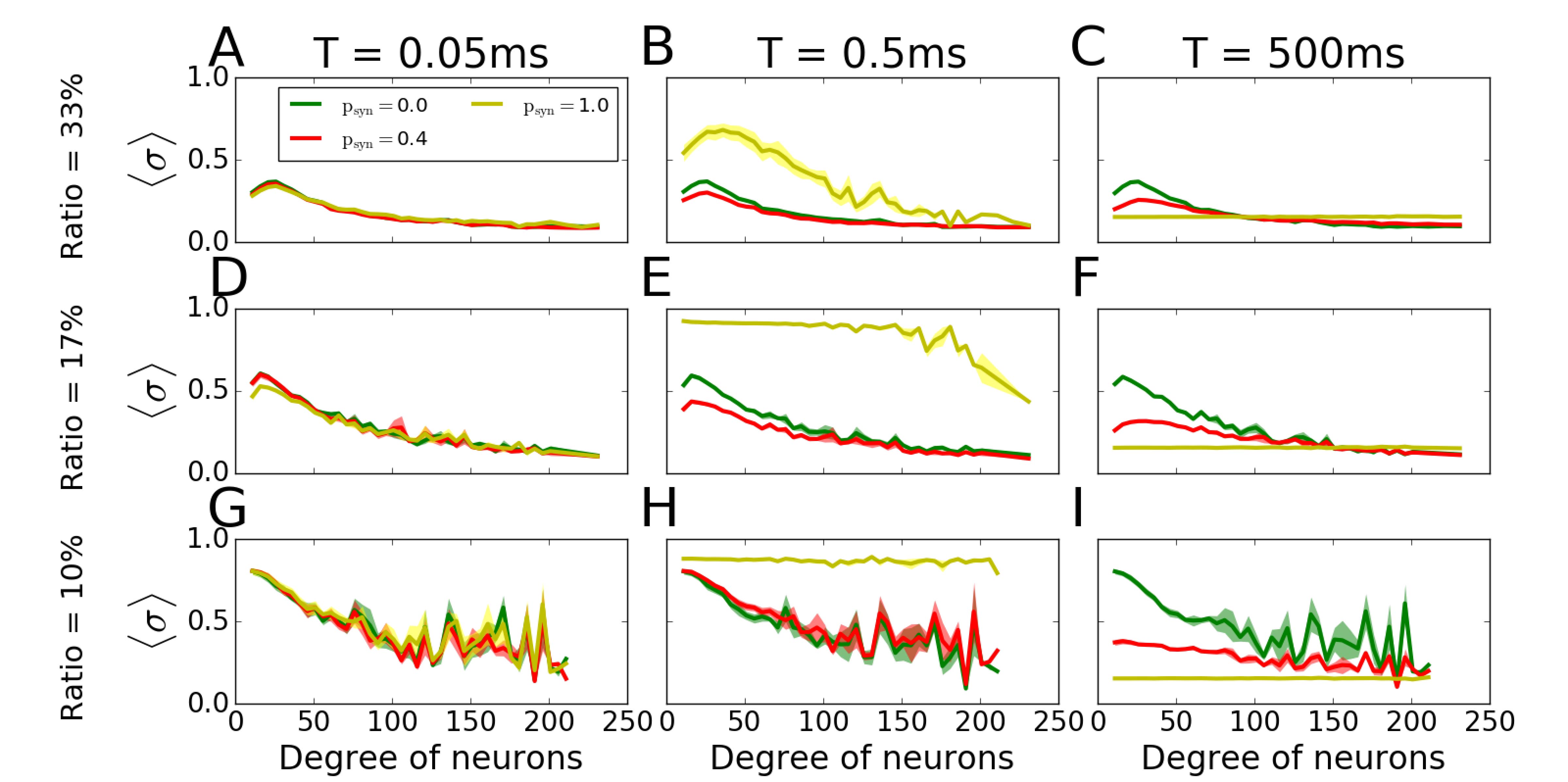

Supplement: FIGURE S5 — Nodal contribution to network-wide MPC as a function of its degree for outgoing networks for different direction ratios and failure recovery time constant T. For T = 0.5 ms, the increase in MPC values of psyn = 1.0 is more pronounced for lower direction ratios. MPCs are averaged over 5 degrees and results are averaged over 5 randomized network realizations. [file Image_5.JPEG]
